# Herpes Zoster Following COVID-19 Vaccination in Long-Term Breast Cancer Survivors

**DOI:** 10.7759/cureus.18418

**Published:** 2021-10-01

**Authors:** Ilaria Toscani, Albina Troiani, Chiara Citterio, Giulia Rocca, Luigi Cavanna

**Affiliations:** 1 Medical Oncology, ASL Piacenza, Piacenza, ITA; 2 ASL Roma 2, General Medicine, Rome, ITA; 3 Onco-Hematology, Hospital Piacenza, Piacenza, ITA; 4 Pharmacy, ASL Piacenza, Piacenza, ITA

**Keywords:** cancer, covid 19, reactivation, zoster, vaccination

## Abstract

Mortality from coronavirus disease 2019 (COVID-19) is higher among patients with cancer. Vaccination represents a cornerstone in overcoming the disease, and vaccine safety needs to be closely assessed. This article discusses two cases of herpes zoster (HZ) following the administration of the BNT162b2 mRNA vaccine in patients who are long-term survivors of breast disease. HZ developed 24 days and two days after the second dose of the vaccine in women aged 81 and 61, respectively. These two patients were breast cancer operated respectively nine and 16 years before; interestingly HZ developed in the same site of previous surgical resection. The patients did not show lymphocytopenia or other signs of immunosuppression and were treated with acyclovir, resulting in the complete resolution of HZ. To our knowledge, these two patients are the first described cases of HZ reactivation following COVID-19 vaccination in cancer survivors.

## Introduction

A novel coronavirus named severe acute respiratory syndrome coronavirus 2 (SARS-CoV-2) emerged in Wuhan, China, in December of 2019; it has since quickly spread and caused a global pandemic [1-3]. SARS-CoV-2 is a highly contagious respiratory pathogen and causes a disease that has been termed the coronavirus disease 2019 (COVID-19). Clinical experience thus far indicates that COVID-19 is highly heterogeneous, ranging from being asymptomatic and mild to being severe and capable of causing death [4-5]. Additionally, COVID-19 has been associated with various cutaneous manifestations, such as: skin eruptions, diffuse erythematous eruptions, widespread urticarial, and chickenpox-like vesicles [6-7].

BNT162b2 and mRNA-1273 area lipid nanoparticles-encapsulated messenger RNA (mRNA)-based vaccines were approved and recommended by the United States Food and Drug Administration and the European Medicine Agency to help mitigate the spread of COVID-19 disease [8-9].

Among the most commonly reported adverse effects of these vaccines are injection site pain, fever, headache, nausea, and vomiting. Herpes zoster (HZ) has been reported in 1.3% of patients vaccinated against COVID-19; however, to our knowledge, none of these patients had history of previous breast cancer [10].

This report describes two cases of the varicella zoster virus (VZV) first reactivating closely after vaccination against SARS-CoV-2 with the BNT162b2 mRNA vaccine. Two women with a history of breast cancer were observed in the Oncology-Hematology Department of Piacenza’s Hospital, northern Italy. Both patients were vaccinated with two doses of the BNT162b2 mRNA vaccine.

## Case presentation

Case 1

**Figure 1:**
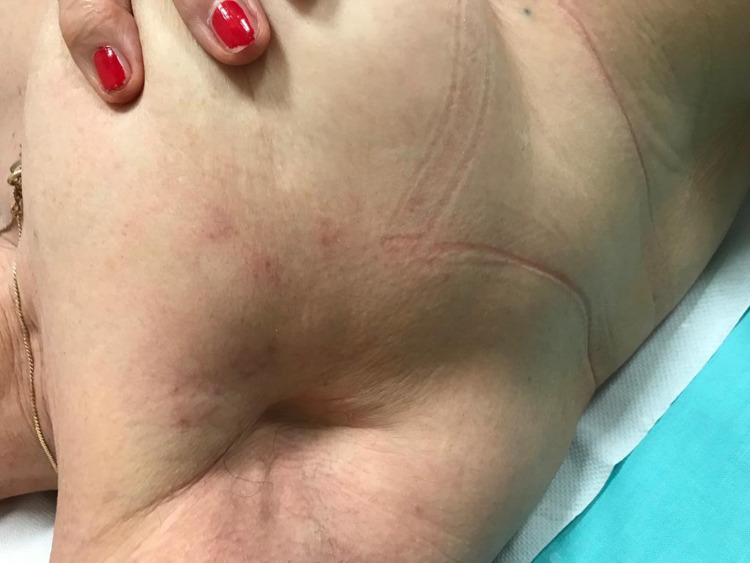
Case 1.

An 84-year-old woman with a history of right breast cancer was treated with surgery (quadrantectomy, QUART) in November 2011, adjuvant chemotherapy, subsequent radiotherapy, and hormone therapy (this one until 2016). The patient had no other relevant copathologies that could increase the risk of HZ. She was presented with the first episode of HZ after COVID-19 vaccination. She received the first dose of the BNT162b2 mRNA vaccine on February 24, 2021, and was injected with the second dose on March 17, 2021. On April 10, 2021, the patient developed right back pain, vesicular skin rashes, and pruritus on the right breast in the area where she was treated with QUART; these findings were all consistent with HZ as seen in Figure 1. She received antiviral therapy and was administered acyclovir at a dosage of 800 mg, delivered four times a day for a period of 10 days. All her symptoms were resolved after this period.

Case 2

**Figure 2:**
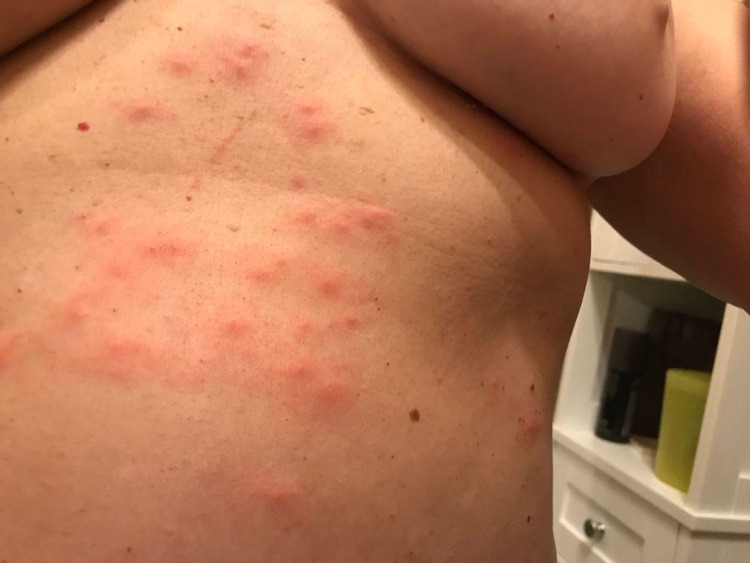
Case 2.

A 61-year-old woman with a history of left breast cancer treated with surgery (radical mastectomy) in August 2005, adjuvant chemotherapy, radiotherapy, and hormone therapy, without other pathologies, presented with the first episode of HZ after COVID-19 vaccination. She received the first dose of the BNT162b2 mRNA vaccine on May 28, 2021 and the second dose on July 2, 2021, with both shots administered to her right arm. Two days after the second dose, the patient observed an increase in the volume of a right supraclavicular lymph node and developed red wheals and pruritus on her left hemithorax and in the area previously operated for left breast cancer; these symptoms were consistent with VZV reactivation as seen in Figure 2. She received antiviral therapy and was administered acyclovir at a dosage of 800 mg, delivered four times a day for a period of 10 days. As in Case 1, her symptoms resolved after this period.

The characteristics of each patient are summarized in Table 1.

**Table 1:** Characteristics of the reported cases of VZV reactivation following vaccination against SARS-CoV-2. CMF, cyclophosphamide + methotrexate + 5 fluorouracil; ER, estrogen receptor; FEC, fluorouracil + epirubicin + cyclophosphamide; HER2, human epidermal growth factor receptor 2; IDC, invasive ductal carcinoma; Ki67, proliferation marker protein; PgR, progesterone receptor; QUART, quadrantectomy; VZV, varicella zoster virus; HZ, herpes zoster

Patient	1	2
Age	84	61
Sex	Female	Female
Medical conditions	Hypertension	No relevant copathologies
Usual medications	None	None
Type of previous breast cancer	IDC	IDC
Side	Right	Left
Surgery	QUART, 23-Nov-2011	Mastectomy, 18-Aug-2005
Stage	pT2a pN2a	pT2 pN1
Grade	2	3
Biology	ER>95%, PgR 30%, Ki67 40%, HER2 score 0	ER 29%, PgR 88%, Ki67 40%, HER2 score 0
Adjuvant radiotherapy	Yes	Yes
Adjuvant chemotherapy	Yes, CMF (six cycles)	Yes, FEC (six cycles)
Adjuvant hormone therapy	Yes, Letrozolo for five years	Yes, Letrozolo + Decapeptyl for five years
Vaccine administered	BNT162b2	BNT162b2
Onset of VZV reactivation (days after second dose)	24	2
VZV reactivation site	Right breast	Left hemithorax
Prior history of HZ	No	No
Other symptoms and adverse reactions	Local pain at administration site	Local pain at administration site

## Discussion

Patients with cancer constitute a vulnerable population at higher risk of contracting severe COVID-19 due to their poor general conditions and systemic immunosuppressive states caused by cancer and by anticancer treatment [11]. When infected by SARS-CoV-2, patients who are cancer survivors or receiving active anticancer treatment have a markedly elevated risk of admission to ICUs, intubation, and death [12]. However, patients with cancer were excluded from major registration trials on SARS-CoV-2 vaccines [13]. A recent meta-analysis and systematic review report data on the efficacy and safety of mRNA COVID-19 vaccines in cancer patients, with most centering on active anticancer treatment, and no studies describing the incidence of HZ following COVID-19 vaccination were found [14].

Varicella zoster virus is a neurotropic virus that remains latent in dorsal-root or cranial-nerve ganglia after primary infection. HZ is attributed to the reactivation of VZV due to risk factors, including increasing age (HZ is particularly common among adults ≥ 50 years of age), immunocompromised conditions such as cancer itself and cancer treatments (previous and ongoing), X-ray irradiation, trauma, and stress [15]. Reactivation of other diseases has also been described in immunocompetent patients with COVID-19 [16] and after many studies on vaccinations, these diseases include: hepatitis A, rabies, and influenza; these findings suggest a vaccine-induced immunomodulation [17].

A clear increase in the risk of HZ has been demonstrated in many solid cancers and there seems to be a substantial heterogeneity in the association according to the type of malignancy. The largest risk of HZ was observed in hematological malignancies and central nervous system cancer, while oral, esophageal, stomach, colorectal, lung, breast, ovarian, prostate, kidney, and bladder cancers were associated with 10%-50% increases in odds of HZ [18].

There are a increasing number of case reports about the development of HZ following the administration of mRNA-based COVID-19 vaccines [10], particularly in patients with autoimmune inflammatory rheumatic diseases such as rheumatoid arthritis, spondyloarthropathies, connective tissue diseases, vasculitis, and myositis [10]. The two cases reported here are the first observations of long-term breast cancer survivors who developed HZ closely following BNT162b2 mRNA vaccination against COVID-19.

The potential mechanisms that might explain the pathogenic link between mRNA-COVID19 vaccination and HZ reactivation are related to the stimulation of innate immunity through toll-like receptors 3,7 by mRNA-based vaccines [19] or to a transient lymphocytopenia that occurs after vaccination, akin to that in the COVID-19 disease [16]. Certainly, we need more studies to investigate the particular mechanism behind the reactivation of VZV in a similar population and identify the risk factors.

## Conclusions

To the best of the researchers’ knowledge, this report represents the first described cases of HZ following COVID-19 vaccine in cancer patients. It must be emphasized that the two patients were not on active anticancer treatment and were without clinical or biological signs of immunosuppression. Furthermore, the cancers of the patients examined in cases 1 and 2 were treated nine years ago and 16 years ago, respectively. These patients did not relapse and did not show lymphocyte penia or other signs of immunosuppression. Interestingly HZ in the two patients developed in the same site of previous surgical resection as a “locus minoris resistentiae” (a body region more vulnerable than other). Certainly, the mass mRNA COVID-19 vaccination campaigns conducted on a global scale will lead to the emergence of a larger number of cases with VZV reactivation, also in cancer patients, and will facilitate further research into the underlying pathogenetic mechanisms.
